# Iron-Deficiency Anemia in Women of Reproductive Age in Urban Areas of Quetta District, Pakistan

**DOI:** 10.1155/2022/6677249

**Published:** 2022-02-09

**Authors:** Mir Abdul Qadir, Nadeem Rashid, Mohummad Alam Mengal, Muhammad Sharif Hasni, Shahab ud din Kakar, Ghulam Mustafa Khan, Nisar Ahmed Shawani, Imran Ali, Irfan Shahzad Sheikh, Nasimullah Khan

**Affiliations:** ^1^Center for Advanced Studies in Vaccinology and Biotechnology (CASVAB), University of Balochistan, Quetta, Pakistan; ^2^Institute of Biochemistry, University of Balochistan, Quetta 87300, Pakistan; ^3^Department of Zoology, University of Balochistan, Quetta 87300, Pakistan; ^4^Department of Chemistry, University of Balochistan, Quetta 87300, Pakistan; ^5^Department of Pharmacy, University of Balochistan, Quetta 87300, Pakistan; ^6^School of Life Science and Engineering, Southwest University of Science and Technology, Mianyang, China; ^7^Institute of Public Health, Quetta 87300, Pakistan

## Abstract

Anemia is associated with poor health outcomes, and the prevalence of anemia is a significant public health indicator for both developed and developing countries. Iron-deficiency anemia (IDA) is the most common type of anemia, which often develops during pregnancy. A cross-sectional study was conducted in the urban areas of Quetta city among the women of reproductive age (15-49 years) to update the status of IDA in the region. The study participants (*n* = 216) were selected on a random basis, and the samples were further distributed by age. Overall, 75% of females were nonanemic, and among those that were anemic, 2% were severe, 13% were moderately, and 10% were mildly anemic. Among the IDA-affected women, 83% were non-pregnant. Age-wise distribution of IDA revealed no significant difference among different age groups, but numerically higher observations were recorded in the age groups of less than 30 years. The highest number of moderately IDA-affected women (15%) were in the age group 15-19 with the following IDA indicating parameters: hemoglobin 9.64 g/dl, mean corpuscular volume 63.11 fl, mean corpuscular hemoglobin 20.40%, red cell distribution 19.28%. This study will be beneficial for illustrating the requirement and the development of a program to raise extended awareness in the Quetta communities to overcome the negative health effects of IDA on the female population.

## 1. Introduction

Anemia, which is a deficiency of red blood cells/hemoglobin in the blood, is a worldwide health problem, with a prevalence of 9% in developed countries and 43% in developing countries [1, 2]. Anemia can happen at any stage of life, and it affects the quality of life (especially in women) [3]. Especially among the vulnerable, this may increase the risk of impaired cognitive and physical development and might also lead to an increase in mortality and morbidity [4]. The etiology of anemia is multifactorial, which can be nutritional, hereditary, or an outcome of infectious and chronic diseases ([5, 6]). Along with these socioeconomic and demographic factors, environmental pollutants, autoimmunity, and malabsorption of nutrients are also predisposing factors of anemia [5].

The most common type of anemia is iron-deficiency anemia (IDA) [7], with the World Health Organization reporting that among two billion anemic people, 50% were IDA affected [8]. A long-term negative iron imbalance or the poor bioavailability/absorption of ingested iron may result in IDA [9]. Iron deficiency usually progresses gradually, and most of the time there are no obvious clinical symptoms until the anemia becomes severe [10, 11]. IDA leads to decreased appetite, which further increases anemia. Iron deficiency causes poor motor and sensory system functioning and delay in cognitive and physical development leading to decreased work capacity of individuals, which in turn affects the development of the country [12, 13].

Quetta is the provincial capital city with almost 19% residents of the total population of in the province of Balochistan, Pakistan, with a fast population growth rate and severe imbalances in diet consumption [14]. This study was designed to update the status of IDA in women of reproductive age (15-49 years) living in the urban area of the Quetta district.

## 2. Material and Methods

In this cross-sectional study, a total of 216 samples were selected on a random basis from women of reproductive age in urban areas of the Quetta district in Balochistan, Pakistan. The study was conducted in a timespans of one year from November 01, 2018, to October 31, 2019. The total population of the Quetta district was 2,269,473 of which 1,190,476 were male and 1,078,718 were female. Urban Quetta is comprised of “Halqas,” which denotes an area with 128,618 households. One “Halqa” was randomly selected to conduct the pilot study. Households were selected based on the record of the female population maintained by the Lady Health Worker (LHW) program.

### 2.1. Sampling Procedure

The women were enlisted from the register of LHWs maintained under the supervision of the In-charge of the respective Basic Health Unit and were serially numbered. Using this as the sampling frame, 216 women of reproductive age (15-49 years) were selected by simple random sampling.

### 2.2. Data Collection

A pretested structured Performa was used to collect the information regarding IDA in the women of reproductive age group 15-49 from the urban area of the Quetta district. The data from the randomly selected women were collected by house-to-house visits with the help of LHWs of the study area. Information regarding variables such as pregnancy history and blood sampling for hematology testing were collected by trained clinical staff. The hematology variables of hemoglobin level (Hb gram/deciliter), mean corpuscular volume (MCV), and red cell distribution width (RDW%) were determined using a cell counter (CELL-DYN Emerald 09H39-01, France).

### 2.3. Ethical Approval

Permission to conduct the study was obtained from the ethical committee of CASVAB, University of Balochistan. During the actual experiment, the purpose of the study was explained to the head of the family and thereafter to the respondent individually, and the consent was obtained.

### 2.4. Data Analysis

Data analysis was done using SPSS version 20.00 (Chicago, IL, USA). Frequencies, percentages, mean, and standard deviation were applied for the descriptive analysis. One-way analysis of variance (ANOVA) was carried out to compare different categories of the independent variables. The *P* value of 0.05 (*P* < 0.05) was taken as the level of significance.

## 3. Results

### 3.1. Status of IDA in Women (15-49 years)

An overview of the status of IDA in 216 randomly selected women of reproductive age (15-49 years) in urban areas of Quetta is shown in Table 1. Overall, 75% were nonanemic, and 25% of the study participants were anemic, whilst 2% were severe, 13% were moderately, and 10% were mildly anemic. Among these, 17% were pregnant at the time of study.

### 3.2. Frequency (%) Distribution of IDA-Positive Women by Age

An overall assessment of IDA-positive women based on the age group is presented in Table 2. All the age groups attributed nonsignificant association (*P* > 0.05) with each other regarding IDA positivity. Based on total observations of a particular age group, the highest number (38.7%) of IDA-positive women were recorded in the age group 15-19 years, whilst the lowest (10.2%) was in the age group 45-49 years. The data pattern of IDA-positive women based on total observations revealed a similar trend that is, the highest (5.6%) in the age group 15-19 years and the lowest (1.4%) in the age group 45-49 years.

### 3.3. Hematology Data (Mean ± SD) for IDA-Positive Women/Age

Table 3 shows the mean hemoglobin, MCV, MCH, and RDW values of IDA-positive women in different age groups. The numerically lowest mean Hb (9.64 ± 1.79), MCV (62.77 ± 4.63), and MCH (20.40 ± 2.96) values, and the highest RDW (19.40 ± 2.72) percentage was observed in the age group 15-19 years.

### 3.4. Distribution of the severity of IDA in affected women/age under WHO criteria

Table 4 shows the severity of IDA-positive women of different age groups according to the criteria of the World Health Organization. Based on hemoglobin values, the highest prevalence of mildly and moderately affected IDA-positive women was observed in the age groups 30 to 34 years and 15 to 19 years, respectively, whereas severely affected IDA-positive women were evenly distributed in the age groups 15-19 years, 30-34 years, and 40-49 years.

## 4. Discussion

IDA affecting women of reproductive age is increasing throughout the world, especially in developing countries. The findings of the present study revealed that overall 25% of women were IDA affected, and among these, a majority were not pregnant at the time of the study. Similarly, Vibhute et al. [15] and Bharati et al. [16] reported that a higher number of nutritionally anemic women of reproductive age were not pregnant at the time of their studies. Whilst anemia can affect any age group [17], women of reproductive age with hectic schedules, erratic mealtimes, and long working hours are more vulnerable [15, 18]. Nutritional anemia due to iron deficiency is a serious public health problem in many parts of the world. Arabyat et al. [19], Machado et al. [20], [17]); Ganapathi and Kumar [21] and Chandyo et al. [22] reported IDA at the rate of 37.3% in Jordan, 12.3% in Brazil, 23.2% in Ethiopia, 24% in Uganda, 53.3% in India, and 12% in Nepal. The World Health Organization reported a 25% prevalence of anemia worldwide during the years 1993-2005, and it is generally assumed that 50% of anemia cases are due to iron deficiency [23]. The differences in the prevalence of anemia among women of reproductive age are multifactorial and vary by geographic region and genetic background [24]. In developing countries, poverty, malnutrition, socioeconomic differences, cultural and dietary pattern, poor literacy ratio, and lack of awareness in the population are the key factors influencing IDA [15, 17]. The risk factors could be further elaborated as poor diet quality, low dietary intake of iron, poor absorption of iron from diets high in phytate or phenolic compounds, impaired immune function, lower BMI, menstruation, low consumption of meat, high intake of tea, blood and intestinal parasitic infection, and consumption of nonfood items such as clay (Pica) [9, 25–28].

The results of the present study concerning age-wise assessment of IDA showed no significant (*P* > 0.05) association among age groups. However, the IDA-positive percentage was relatively higher in the age groups less than 25 years. In this respect, Lilare and Durgesh [29], Bharati et al. [30] and Bharati at al. [16] have reported a higher prevalence of anemia in lower age groups (less than 25 years) in India. This might be because early childbearing is very common in India and half of childbearing aged women had a birth before they were 20 years old [25].

Hematological assessment of IDA-positive women revealed no significant (*P* > 0.05) difference among different age groups. However, numerically lower Hb, MCV, and MCH values and higher RDW values were observed in the age group 15-19 years. Similar findings were observed by Tkaczszyn et al. [31], who reported IDA patients with lower Hb (11.3 ± 1.1 g/dl), MCV (88.7 ± 6.2 fl) and MCH (28.2 ± 2.5 pg) values and higher red cell distribution width (RDW) RDW (16 ± 2%) values than the normal range (Hb < 12 g/dl, MCV83.6-97 fl, MCH 27-32 pg, RDW 11.6-14.3%) [32]. Deficiency of the metabolically active trace element iron in the iron stores of body itch, in turn, is influenced by socioeconomic and geographical characteristics and cultural practices; females can use vegetable diet due to poverty and social discrimination and use meat that led to IDA being influenced by dietary habits and dietary patterns [33]. High intake of tea contains phytate, and using vegetable-based diet provides a low amount of bioavailable iron because of the high content of iron absorption inhibitors such as phytate and polyphenols then causing iron-deficiency anemia [6].

In women, loss of iron from the body mostly happens through sloughing of duodenal enterocytes and through menstrual and postpartum blood loss that deprive the circulatory pool of iron that is available for its utilization in target organs resulting in the state of functional iron deficiency, which is measured in the form of hemoglobin (iron is the main ingredient of hemoglobin) [33]. Low red blood cell indices are considered sensitive indicators of decreased iron availability for hematopoietic tissues [34]. A decrease in Hb, MCV, and MCH reflects iron-restricted erythropoiesis in the bone marrow, which reflects a typical IDA presentation [31]. Hematological parameters such as Hb, MCV, MCH, and RDW are directly influenced in IDA, where Hb, MCV, and MCH values decrease, whilst RDW increases [13]. An increase in RDW is a good indicator for early detection of IDA and RDW in combination with MCV, MCH, and Hb evaluation which is a good differential diagnosis tool. In megaloblastic anemia, there could be increased RDW with decreased Hb [35], along with an increase in MCV and a decrease in IDA and MCH [13].

The findings of the present work evaluating the severity of IDA in women according to WHO criteria revealed that the anemia severity was mainly mild followed by moderate and severe. These findings are in agreement with the observations of Le [36], Lilare and Durgesh [29] and Saroshe et al. [12]. However, Verma et al. [3], in a community-based cross-sectional study conducted in Mumbai, India, inferred that the severity of anemia in the IDA affected population was mainly moderate followed by mild and severe. The severity of anemia in women of reproductive age is multifactorial, greatly depending on the socioeconomics, literacy rate, level of awareness, and cultural habits of the population [29]. It is important to recognize that mild-stage anemia is usually asymptomatic and therefore does not attract attention. It is only when anemia advances to moderate and severe stages that symptoms are revealed and the individual seeks therapeutic intervention [34].

## 5. Conclusion

The findings of this study lead to the conclusion that IDA is a major public health problem particularly for women of reproductive age (less than 30 years) living in urban areas. Greater efforts are therefore required to overcome the problem, which may include supplementation of women with iron and folic acid and an awareness campaign regarding IDA not only in the subject community but to the whole population. Furthermore, studies on the said research area will be helpful to further clear the scenario especially in rural areas.

## Figures and Tables

**Table 1:** Overview of the status of IDA in women (15-49 y).

Anemia	Pregnant (%)	Nonpregnant (%)	Total (%)
Non	12	63	75
Mild	2	8	10
Moderate	3	10	13
Severe	0	2	2
Total	17	83	100

**Table 2:** Frequency (%) distribution of IDA-positive women by age.

Age group (years)	A∗	B∗	C∗	D∗
15-19	14.4	38.7	20	5.6
20-24	13.4	27.6	13.33	3.7
25-29	17.6	28.9	18.33	5.1
30-34	16.7	25.0	15	4.2
35-39	15.7	26.5	15	4.2
40-44	12.0	30.8	13.33	3.7
45-49	10.2	13.7	5	1.4

A∗ Frequency on the basis of total observations including all age groups. B∗ IDA-positive frequency on the basis of total observations in the respective age group. C∗ IDA-positive frequency on the basis of total positive observations including all age groups. D∗ IDA-positive frequency on the basis of total observations including all age groups.

**Table 3:** Hematology data (mean ± SD) for IDA-positive women/age.

Age group (years)	Hb (g/dl)	MCV (fl)	MCH	RDW (%)
15-19	9.64 ± 1.67	63.11 ± 8.54	20.40 ± 2.96	19.28 ± 2.86
20-24	10.26 ± 1.13	69.33 ± 15.17	22.66 ± 5.69	19.01 ± 5.20
25-29	10.41 ± 1.16	69.08 ± 4.46	22.69 ± 1.77	17.59 ± 2.64
30-34	10.74 ± 1.26	67.20 ± 11.28	21.63 ± 4.29	17.83 ± 3.84
35-39	10.91 ± 0.71	75.20 ± 12.46	24.41 ± 4.37	17.74 ± 4.56
40-44	10.21 ± 1.75	73.22 ± 18.50	21.60 ± 5.88	19.15 ± 4.63
45-49	10.50 ± 0.34	71.73 ± 6.87	24.13 ± 2.22	15.80 ± 2.30
Over all	10.35 ± 1.30	69.53 ± 12.05	22.35 ± 4.20	18.28 ± 3.84

**Table 4:** Distribution of the severity of IDA in affected women/age under WHO criteria.

Age group (years)	Mild		Moderate		Severe	
	A∗	B∗	A∗	B∗	A∗	B∗
15-19	8.7	3.3	26.5	15.0	33.3	1.7
20-24	13.0	5.0	14.7	8.3	0.0	0.0
25-29	13.0	5.0	23.5	13.3	0.0	0.0
30-34	26.1	10.0	5.9	3.3	33.3	1.7
35-39	21.7	8.3	11.8	6.7	0.0	0.0
40-44	17.4	6.7	8.8	5.0	33.3	1.7
45-49	0.0	0.0	8.8	5.0	0.0	0.0

A∗ Percentage on the basis of severity (mild/moderate/severe) of IDA-positive women. B∗ Percentage on the basis of total IDA-positive women.

## Data Availability

The data will be make available on request.

## References

[1] Hussain S., Habib A., Najmi A. K. (2019). Anemia prevalence and its impact on health-related quality of life in Indian diabetic kidney disease patients: evidence from a cross-sectional study. *Journal of Evidence-Based Medicine*.

[2] Savarese G., Jonsson Å., Hallberg A. C., Dahlström U., Edner M., Lund L. H. (2020). Prevalence of, associations with, and prognostic role of anemia in heart failure across the ejection fraction spectrum. *International Journal of Cardiology*.

[3] Verma R., Kharb M., Deswal S., Arora V., Kamboj R. (2014). Prevalence of anaemia among women of reproductive age group in a rural block of Northern India. *Indian Journal of Community Health*.

[4] Abuaisha M., Itani H., El Masri R., Antoun J. (2020). Prevalence of iron deficiency (ID) without anemia in the general population presenting to primary care clinics: a cross-sectional study. *Postgraduate medicine*.

[5] Al-Alimi A. A., Bashanfer S., Morish M. A. (2018). Prevalence of iron deficiency anemia among university students in Hodeida Province, Yemen. *Anemia*.

[6] Percy L., Mansour D., Fraser I. (2017). Iron deficiency and iron deficiency anaemia in women. *Best practice & research Clinical obstetrics & gynaecology*.

[7] Nouri A., Matur A., Pennington Z. (2020). Prevalence of anemia and its relationship with neurological status in patients undergoing surgery for degenerative cervical myelopathy and radiculopathy: a retrospective study of 2 spine centers. *Journal of Clinical Neuroscience*.

[8] Mehrotra M., Yadav S., Deshpande A., Mehrotra H. (2018). A study of the prevalence of anemia and associated sociodemographic factors in pregnant women in Port Blair, Andaman and Nicobar Islands. *Journal of Family Medicine and Primary Care*.

[9] Nasir B. B., Fentie A. M., Adisu M. K. (2020). Adherence to iron and folic acid supplementation and prevalence of anemia among pregnant women attending antenatal care clinic at Tikur Anbessa Specialized Hospital, Ethiopia. *Plos One*.

[10] Belisário A. R., de Almeida J. A., Mendes F. G. (2020). Prevalence and risk factors for albuminuria and glomerular hyperfiltration in a large cohort of children with sickle cell anemia. *American Journal of Hematology*.

[11] Hisa K., Haruna M., Hikita N. (2019). Prevalence of and factors related to anemia among Japanese adult women: secondary data analysis using health check-up database. *Scientific Reports*.

[12] Saroshe S., Pandey D., Dixit S., Shukla H., Tiwari S. (2014). Estimation of prevalence of anemia using WHO hemoglobin color scale among non pregnant females of urban slum. *Global Journal of Medicine and Public Health*.

[13] Sazawal S., Dhingra U., Dhingra P. (2014). Efficiency of red cell distribution width in identification of children aged 1-3 years with iron deficiency anemia against traditional hematological markers. *BMC Pediatrics*.

[14] Huda S. U. (2014). Determinants of population growth in Pakistan. *International Journal of Endorsing Health Science Research*.

[15] Vibhute N. A., Shah U., Belgaumi U., Kadashetti V., Bommanavar S., Kamate W. (2019). Prevalence and awareness of nutritional anemia among female medical students in Karad, Maharashtra, India: a cross-sectional study. *Journal of Family Medicine and Primary Care*.

[16] Bharati P., Som S., Chakrabarty S., Bharati S., Pal M. (2008). Prevalence of anemia and its determinants among nonpregnant and pregnant women in India. *Asia Pacific Journal of Public Health*.

[17] Obai G., Odongo P., Wanyama R. (2016). Prevalence of anaemia and associated risk factors among pregnant women attending antenatal care in Gulu and Hoima Regional Hospitals in Uganda: a cross sectional study. *BMC Pregnancy and Childbirth*.

[18] Retnakumar C., Chacko M., Ramakrishnan D., George L. S., Krishnapillai V. (2020). Prevalence of anemia and its association with dietary pattern among elderly population of urban slums in Kochi. *Journal of Family Medicine and Primary Care*.

[19] Arabyat R., Arabyat G., al Taani G. (2019). Prevalence and risk factors of anaemia among ever-married women in Jordan. *Eastern Mediterranean Health Journal*.

[20] Machado I. E., Malta D. C., Bacal N. S., Rosenfeld L. G. M. (2019). Prevalence of anemia in Brazilian adults and elderly. *Revista Brasileira de Epidemiologia*.

[21] Ganapathi K. C., Kumar K. S. (2017). A cross-sectional study of anemia among women of reproductive age group (15-49 years) in a rural population of Tamil Nadu. *International Journal of Medical Science and Public Health*.

[22] Chandyo R. K., Strand T. A., Ulvik R. J. (2007). Prevalence of iron deficiency and anemia among healthy women of reproductive age in Bhaktapur, Nepal. *European Journal of Clinical Nutrition*.

[23] De Benoist B., McLean E., Egli I., Cogswell M., WHO (2008). *Worldwide Prevalence of Anaemia1993–2005*.

[24] Zhang X., He Y., Xie X., Ji M., Ma X., Yu Z. (2017). Distribution of hemoglobin and prevalence of anemia in 10 ethnic minorities in China: a population-based, cross-sectional study. *Medicine*.

[25] Asres Y., Yemane T., Gedefaw L. (2014). Determinant Factors of Anemia among Nonpregnant Women of Childbearing Age in Southwest Ethiopia: A Community Based Study. *International Scholarly Research Notices*.

[26] Baig-Ansari N., Badruddin S. H., Karmaliani R. (2008). Anemia prevalence and risk factors in pregnant women in an urban area of Pakistan. *Food and Nutrition Bulletin*.

[27] Bhargava A., Bouis H. E., Scrimshaw N. S. (2001). Dietary intakes and socioeconomic factors are associated with the hemoglobin concentration of Bangladeshi women. *The Journal of Nutrition*.

[28] Manzoor M., Manzoor M., Ahmed Q., Ahmed S., Manzoor S. (2017). Frequency and causes of iron deficiency anemia in patients visiting gynae outdoor unit: an institutional based study. *Punjab University Journal of Zoology*.

[29] Lilare R. R., Sahoo D. P. (2017). Prevalence of anaemia and its epidemiological correlates among women of reproductive age group in an urban slum of Mumbai. *International Journal of Community Medicine and Public Health*.

[30] Bharati S., Pal M., Som S., Bharati P. (2015). Temporal trend of anemia among reproductive-aged women in India. *Asia Pacific Journal of Public Health*.

[31] Tkaczyszyn M., Jankowska E. A. (2017). Iron deficiency and red cell indices in patients with heart failure: reply. *European Journal of Heart Failure*.

[32] Arbiol-Roca A., Imperiali C. E., Montserrat M. M. (2018). Reference intervals for a complete blood count on an automated haematology analyser Sysmex XN in healthy adults from the southern metropolitan area of Barcelona. *Ejifcc*.

[33] Negi P. C., Dev M., Paul P. (2018). Prevalence, risk factors, and significance of iron deficiency and anemia in nonischemic heart failure patients with reduced ejection fraction from a Himachal Pradesh heart failure registry. *Indian Heart Journal*.

[34] Li H., Xiao J., Liao M. (2020). Anemia prevalence, severity and associated factors among children aged 6–71 months in rural Hunan Province, China: a community-based cross-sectional study. *BMC Public Health*.

[35] Briggs C. (2009). Quality counts: new parameters in blood cell counting. *International Journal of Laboratory Hematology*.

[36] Le C. H. H. (2016). The prevalence of anemia and moderate-severe anemia in the US population (NHANES 2003-2012). *PLoS One*.

